# Evaluation of a phased pneumococcal conjugate vaccine introduction in Mongolia using enhanced pneumonia surveillance and community carriage surveys: a study protocol for a prospective observational study and lessons learned

**DOI:** 10.1186/s12889-019-6639-y

**Published:** 2019-03-21

**Authors:** S. F. La Vincente, C. von Mollendorf, M. Ulziibayar, C. Satzke, L. Dashtseren, K. K. Fox, E. M. Dunne, C. D. Nguyen, J. de Campo, M. de Campo, H. Thomson, G. Surenkhand, S. Demberelsuren, S. Bujinlkham, L. A. H. Do, D. Narangerel, T. Cherian, T. Mungun, E. K. Mulholland

**Affiliations:** 1 Pneumococcal Research Group, Murdoch Children’s Research Institute, Melbourne, Australia; 2 Department of Paediatrics, The University of Melbourne, Melbourne, Australia; 3 National Center of Communicable Diseases, Ulaanbaatar, Mongolia; 4 Department of Microbiology and Immunology, The University of Melbourne at the Peter Doherty Institute for Infection and Immunity, Melbourne, Australia; 5 World Health Organization Western Pacific Regional Office, Manila, Philippines; 6 Department of Radiology, The University of Melbourne, Melbourne, Australia; 7 World Health Organization Country Office, Ulaanbaatar, Mongolia; 8 Ministry of Health, Ulaanbaatar, Mongolia; 9 Department of Immunization, Vaccines and Biologicals, World Health Organization, Geneva, Switzerland; 10 Telethon Kids Institute, Perth, Australia; 11 London School of Hygiene and Tropical Medicine, London, UK

**Keywords:** *Streptococcus pneumoniae*, Pneumococcal conjugate vaccine, Mongolia, Nasopharyngeal carriage, Pneumonia, Surveillance, Vaccine impact evaluation

## Abstract

**Background:**

*Streptococcus pneumoniae* causes substantial morbidity and mortality among children. The introduction of pneumococcal conjugate vaccines (PCV) has the potential to dramatically reduce disease burden. As with any vaccine, it is important to evaluate PCV impact, to help guide decision-making and resource-allocation. Measuring PCV impact can be complex, particularly to measure impact on one of the most common and significant diseases caused by the pneumococcus, namely pneumonia. Here we outline the protocol developed to evaluate the impact of 13-valent PCV (PCV13) on childhood pneumonia in Mongolia, and a number of lessons learned in implementing the evaluation that may be helpful to other countries seeking to undertake pneumonia surveillance.

**Methods:**

From 2016 PCV13 was introduced in a phased manner into the routine immunisation programme with some catch-up by the Government of Mongolia. We designed an evaluation to measure vaccine impact in children aged 2–59 months with hospitalised radiological pneumonia as a primary outcome, with secondary objectives to measure impact on clinically-defined pneumonia, nasopharyngeal carriage of *S. pneumoniae* among pneumonia patients and in the community, and severe respiratory infection associated with RSV and/or influenza. We enhanced an existing hospital-based pneumonia surveillance system by incorporating additional study components (nasopharyngeal swabbing using standard methods, C-reactive protein, risk factor assessment) and strengthening clinical practices, such as radiology as well as monitoring and training. We conducted cross-sectional community carriage surveys to provide data on impact on carriage among healthy children.

**Discussion:**

Establishing a robust surveillance system is an important component of monitoring the impact of PCV within a country. The enhanced surveillance system in Mongolia will facilitate assessment of PCV13 impact on pneumonia, with radiological confirmed disease as the primary outcome. Key lessons arising from this evaluation have included the importance of establishing a core group of in-country staff to be responsible for surveillance activities and to work closely with this team; to be aware of external factors that could potentially influence disease burden estimates; to be flexible in data collection processes to respond to changing circumstances and lastly to ensure a consistent application of the pneumonia surveillance case definition throughout the study period.

## Background

*Streptococcus pneumoniae* (the pneumococcus) is a major cause of illness and mortality among children worldwide, causing otitis media, meningitis, and pneumonia [1]. Pneumococcal conjugate vaccines (PCV) are widely used in high-income countries, and in recent years there has been increased introduction among low- and middle-income countries (LMICs).

With any new vaccine introduction, countries seek evidence of PCV impact in their own population to guide decision-making and allocation of limited health resources. In assessing the impact of PCV, many countries have used invasive pneumococcal disease (IPD) as the primary endpoint [2–7], however this endpoint can be relatively rare. In such settings, large impact studies (usually in the tens of thousands) are needed to account for year-to-year variation in case numbers and changes in serotype distribution. IPD relies on robust sample collection (prior to antibiotic use) and diagnostic clinical microbiology capacity, which is often not available in resource-limited settings. Moreover, restricting the evaluation of PCV impact to IPD misses the opportunity to measure vaccine impact on non-bacteraemic pneumonia, one of the most common and significant illnesses caused by the pneumococcus.

Pneumonia is the most common cause of childhood death globally. Pneumococcal pneumonia contributes substantially to the most severe and often fatal forms of pneumonia [8]. There are few data on the impact of PCV on pneumonia in LMICs, due in part to inherent challenges in diagnosing pneumonia and establishing a cause-specific aetiology.

Nasopharyngeal pneumococcal carriage is a precursor to pneumococcal disease [9]. PCVs reduce vaccine-type (VT) serotype carriage while overall pneumococcal carriage is only slightly decreased or unchanged due to serotype replacement. Serotype replacement has been documented following the introduction of PCV [10, 11] and it is critical to consider and monitor as part of long-term vaccine impact. Isolating a pneumococcus from a nasopharyngeal swab in a child with pneumonia does not necessarily indicate a pneumococcal aetiology. However a reduction in the detection of VT serotypes over time will reflect vaccine effectiveness in preventing pneumococcal pneumonia episodes due to those serotypes [12].

Mongolia is a land-locked country in Central Asia. Nearly half of its 3 million people live in the capital, Ulaanbaatar, with the remainder being dispersed over a vast geographic area. In Mongolia, pneumonia is one of the most common causes of mortality among children, accounting for 25% of deaths among children under the age of 5 outside of the neonatal period [13]. The Government of Mongolia (GoM) decided to introduce the 13-valent PCV (PCV13) and to monitor its impact. A collaboration was established between the GoM, the World Health Organization (WHO) and the Murdoch Children’s Research Institute (MCRI) to evaluate the impact of PCV introduction in Mongolia. In the context of relatively low rates of culture-confirmed IPD, and an existing hospital-based surveillance system enrolling cases of pneumonia, we selected pneumonia as the primary endpoint. Here we describe the methodological approach taken to develop prospective hospital-based surveillance for measurement of vaccine impact on pneumonia, the use of community carriage surveys and some of the key challenges and lessons learnt in this ongoing surveillance programme. We believe that this information will benefit other resource-limited countries seeking to establish pneumonia surveillance for the measurement of vaccine impact.

## Evaluation objectives

An evaluation plan was developed to use enhanced hospital-based pneumonia surveillance to document the impact of PCV on hospitalised pneumonia in children aged 2–59 months, and cross-sectional carriage surveys of healthy children to measure vaccine impact on community carriage of pneumococci. Additionally, we will evaluate the PCV impact on severe and very severe lower respiratory tract infection (LRTI) cases associated with respiratory syncytial virus (RSV) and influenza virus in a subset of children aged 2–23 months.

This evaluation has been approved by the Human Research Ethics committees of the Mongolian Ministry of Health, the Royal Children’s Hospital (Melbourne) and the World Health Organization Western Pacific Regional Office.

Overall, there are six study objectives:

### Primary objective

1. *To evaluate the impact of PCV13 introduction on the incidence of radiological pneumonia among children aged 2–59 months*

The primary endpoint was selected to capture the major burden of pneumococcal disease, pneumonia, using a standardised WHO definition of radiological pneumonia with increased specificity compared to other pneumonia classifications [14]. In previous clinical trials, radiological pneumonia best discriminated between PCV vaccinated and control groups [15–18], likely due to a higher proportion of pneumococcal cases. An effective PCV program will therefore have a greater impact on this endpoint.

### Secondary objectives

2) *To evaluate the impact of PCV13 introduction on the incidence of severe and very severe pneumonia in children aged 2–59 months*

To measure the full impact of PCV13 on pneumonia the impact on clinical parameters including severe and very severe pneumonia will also be assessed. Although this outcome can lack specificity, it is relevant for settings where childhood pneumonia is diagnosed on clinical parameters, usually based on the presence of cough and raised respiratory rate. Importantly, a randomised controlled trial in The Gambia, evaluating three doses of 9-valent PCV in infants showed a vaccine efficacy of 7% (95% confidence interval (CI) 1–12) against clinical pneumonia, and 12% efficacy (95% CI -9-29) against severe clinical pneumonia (using the earlier Integrated Management of Childhood Illness [IMCI] definition, which included chest indrawing as a sign of severe pneumonia) [19].

3) *To evaluate the impact of PCV13 introduction on the incidence of definite or probable pneumococcal pneumonia in children aged 2–59 months*

In young children, definitive diagnosis of pneumococcal pneumonia is rare, as they do not produce sputum, and very few pneumonia cases are bacteraemic, making blood cultures a highly insensitive test [20]. To address this, our study will incorporate a definition of probable pneumococcal pneumonia [21].

Surveillance cases will undergo routine testing for C-reactive protein (CRP), a non-specific “marker” for disease, and nasopharyngeal swabbing, to enable an assessment of vaccine impact on a subgroup of radiological pneumonia patients that may be considered either probable (elevated CRP, high density pneumococci in the nasopharynx, pathogenic serotypes) or definite (e.g. bacteraemic culture-positive) pneumococcal pneumonia cases.

4) *To evaluate the impact of PCV13 on prevalence of vaccine type (VT) and non-vaccine type (NVT) pneumococci nasopharyngeal carriage in hospitalised children with radiologically confirmed pneumonia*

We will evaluate the change in overall, VT and NVT carriage in hospitalised children with radiologically-confirmed pneumonia following PCV13 introduction. A study conducted in Israel [22] prior to PCV introduction, showed that children admitted with radiologically-confirmed pneumonia were more likely to carry certain serotypes (1, 5, 7F, 9 V, 14, 19A and 22F) compared with healthy controls. We will be able to detect reductions in VT pneumococci in the nasopharynx of pneumonia patients with radiologically confirmed disease, and identify any increases in NVTs following PCV introduction.

5) *To evaluate the impact of PCV13 on prevalence of vaccine type (VT) and non-vaccine type (NVT) pneumococci nasopharyngeal carriage in hospitalised children with severe clinical pneumonia*

We will evaluate the change in overall, VT and NVT carriage in hospitalised children with severe clinical pneumonia following PCV13 introduction. Depending on total annual pneumonia cases admitted, we may focus on subgroups such as hypoxic children, children who are readmitted and children with viral co-infections for testing.

6) *To evaluate the impact of PCV13 on prevalence of VT and NVT pneumococci nasopharyngeal carriage in the community*

It is important to document the impact of PCV13 on both VT and NVT carriage in the community. Reductions in VT carriage in vaccinated children have been associated with reductions in carriage at the population level [23], while both NVT carriage and rates of NVT invasive disease have increased [24]. Currently, data on vaccine impact including serotype replacement from LMICs are limited. In Mongolia we are conducting cross-sectional community carriage surveys to assess nasopharyngeal pneumococcal carriage before introduction of the vaccine and for 2 years post-PCV13 introduction, in a sample of healthy children aged 12–23 months (eligible to be vaccinated) and in a sample of young infants aged 5–8 weeks (too young to be vaccinated).

7) *To evaluate the impact of PCV13 introduction on the incidence of severe LRTI associated with RSV and/or influenza among children aged 2–23 months.*

RSV and influenza, like the pneumococcus, are important causes of childhood pneumonia globally, especially in children less than 2 years of age [25, 26]. Although the interaction between these two viruses and *S. pneumoniae* has been described using in vitro and animal models, clinical data on the impact of PCV on the burden of RSV and/or influenza infections is still scarce. To date only two studies explored the association between PCV use and RSV or influenza infections: a retrospective time series analysis of US hospitalisation data comparing pre- (1992/1993) and post-PCV7 (2008/2009) data [27] and a double-blind, randomized, placebo controlled trial in South Africa exploring the impact of the 9-valent PCV on viral-associated pneumonia [28]. These data suggest a beneficial effect of PCV on pneumonia associated with viruses. The impact of PCV13 on pneumonia associated with RSV and influenza in young children needs to be explored further, particularly in LMICs, where the benefit may be greater given the higher rates of disease and severity.

For this endpoint we use the WHO definition for a severe LRTI case (defined as an acute illness associated with cough or breathing difficulty and fast breathing (respiratory rate > 50) and arterial oxygen saturation (SaO_2_) less than 93% [29]) and polymerase chain reaction (PCR) detection of RSV or influenza virus from a nasopharyngeal swab.

## Research setting

Mongolia has a very well-structured health system with accurate data on the number of under-5 children in every district. The vast majority of healthcare for children is provided through the public health system with few children accessing the private sector. Most pneumonia cases are therefore captured by the public health services in district and tertiary care facilities. Hospital care for children is geographically and financially accessible in Ulaanbaatar, with inpatient care provided free of charge for children. As such, the vast majority of cases of severe pneumonia among age-eligible children residing in the evaluation districts will be captured by the surveillance system. The surveillance system may thus be considered population-based, permitting the calculation of incidence rates.

The GoM identified four of the nine districts of Ulaanbaatar to participate in the evaluation (Songinokhairkhan, Sukhbaatar, Chingeltei and Bayanzurkh). These are four of the largest districts of Ulaanbaatar, together comprising approximately 70% of the city’s population. In 2012, there were 5903 under-5 year old hospital admissions with a discharge diagnosis of pneumonia in the participating districts, from an estimated population of 98,433 (60 episodes per 1000 under-5 population). There is strong seasonal variation in pneumonia incidence, with the peak season occurring in the winter months from October until April. Eighty percent (*n* = 4772) of the 5903 pneumonia cases admitted in 2012 occurred during these 7 months.

The GoM is introducing PCV13 in a phased manner. This approach is consistent with the standard approach taken to new vaccine implementation in the country, and also facilitates rigorous evaluation of impact by allowing for contemporaneous comparison of vaccinated and unvaccinated populations, rather than relying on before and after comparisons alone. The first phase of PCV13 introduction in two of the evaluation districts (Songinokhairkhan and Sukhbaatar, termed “Phase 1 districts”) commenced at the start of June 2016 (Table 1), followed by stepped introduction in the remaining evaluation districts (“Phase 2 districts”). PCV13 was introduced into one Phase 2 district (Bayanzurkh) in June 2017, with introduction into the second Phase 2 district (Chingeltei) and other districts of Ulaanbaatar from March 2018.

**Table 1:** Evaluation timeline

	Phase 1 districts	Phase 2 districts	
	Songinokhairkhan and Sukhbaatar	Bayanzurkh	Chingeltei
April 2015	Enhanced surveillance commences		
	14 months pre-vaccine surveillance	26 months pre-vaccine surveillance	35 months pre-vaccine surveillance
June 2016	Vaccination commences		
July 2017		Vaccination commences	
March 2018			Vaccination commences

PCV13 was implemented as a three-dose schedule, with doses given to infants at two, four and 9 months of age. There was a three-month catch-up campaign at the time of vaccine introduction in 2016 and 2017 in each of the evaluation districts for children up to 23 months of age, who were given two doses of PCV13 (2 months apart) to maximise vaccine impact, alongside vaccine introduction in 2016 and 2017. In 2018 vaccine introduction into Chingeltei and the remaining districts of Ulaanbaatar was done without a catch-up component.

## Materials and methods

### Enhanced hospital-based surveillance of pneumonia in children aged 2–59 months

At the time that evaluation data collection systems were being established, a surveillance system for meningitis, sepsis and pneumonia was in place in most district hospitals in Ulaanbaatar, as part of the WHO-supported Invasive Bacterial Vaccine Preventable Disease (IB-VPD) Surveillance Network initiated in 2007. Under this system, all patients older than 59 days and less than 5 years of age admitted to a participating hospital with suspected meningitis, pneumonia or sepsis are enrolled. Hospital staff members identify admissions that meet the surveillance case definitions, complete a standard case report form (CRF) and collect relevant specimens. Blood cultures are routinely performed in all suspect cases of pneumonia and sepsis. In cases of suspected meningitis, cerebrospinal fluid culture is also performed. Isolates of *H. influenzae* and pneumococci identified through this surveillance system are serotyped. The incidence of IPD as estimated through this surveillance was approximately 20/100,000 children/year. Data on respiratory-related IPD cases will continue to be collected throughout the evaluation; however based on the low rates of invasive respiratory disease pre-vaccination the likelihood of documenting any meaningful impact is very small, hence it is excluded from the study objectives.

Five hospitals are participating in the evaluation, including one district hospital in each of the four districts, plus the tertiary level referral hospital that receives paediatric admissions from across the country, the National Centre for Maternal and Child Health. All five hospitals participating in the PCV impact evaluation are IB-VPD surveillance sites.

From 1st April 2015, the IB-VPD system was enhanced in the five evaluation hospitals to enable evaluation of the impact of PCV13 introduction on pneumonia. The modifications included the following:

#### Case report form

The PCV13 evaluation system CRF expanded on the IB-VPD CRF. The IB-VPD CRF included information on presenting symptoms and signs, previous medication, immunisation history, specimens collected and treatment received. The enhanced surveillance added questions on oxygen saturation and therapy, and an additional risk factor questionnaire.

#### Radiology data

The IB-VPD system is primarily a laboratory network, and hence in the Mongolian IB-VPD system there was not a strong focus on documenting radiology findings for pneumonia cases. If done, radiographic findings were recorded on the surveillance CRF, but films were not retained by the hospital or available to the surveillance system. Fluoroscopy was preferred to x-rays in many of the facilities in Ulaanbaatar, due to the high cost of printing x-ray films. To enhance the surveillance system for the measurement of vaccine impact on radiological pneumonia, there was a major focus on strengthening and standardising the collection and archiving of chest radiographs for all pneumonia cases. This included delivery of training to radiologists and radiographers in current global standards for paediatric chest radiology. Systems and equipment were established to enable radiographs to be digitised and stored for re-reading according to the standardised WHO criteria.

#### Pneumococcal carriage and viral testing

We added nasopharyngeal sampling as part of the enhanced surveillance. A nasopharyngeal swab is collected from all pneumonia cases who consent, to investigate the presence of pneumococci using *lytA* real-time quantitative PCR (qPCR) [30] and molecular serotyping by DNA microarray. Microarray is the most sensitive method for pneumococcal carriage studies [31]. Together, this approach provides quantitative data on all pneumococcal serotypes present within a sample. Additionally, validated qPCR testing for influenza and RSV is performed on selected severe LRTI cases [32–35].

#### Additional blood for CRP testing

The volume of blood taken from pneumonia admissions was increased to be used for CRP testing. At the time that blood is collected for culture (1–6 ml), an additional 0.5 ml of blood is taken in a serum-separating tube and sent for testing at the National Center of Communicable Diseases (NCCD) laboratory.

#### More specific surveillance case definition

The case definition applied in the pre-existing WHO-supported system was highly sensitive, with all children aged 1–4 years eligible for surveillance if they had a respiratory rate of 40 or more breaths per minute. However, many of these patients were not true cases of pneumonia. To reduce the workload for clinicians, and thus make it more feasible for all eligible pneumonia patients to be enrolled with complete data, a more specific case definition was required. Importantly, a more specific case definition also increases the likelihood of being able to measure vaccine impact, by removing cases unlikely to be due to pneumococcus. Under the new case definition, a case (regardless of age) would have a respiratory rate of 50 or more breaths per minute. This cut-off has a higher specificity than the 40 or more breaths cut-off [36].

Review of a sample of medical records revealed that a number of children had low oxygen saturation without an elevated respiratory rate. Given the importance of hypoxia as a sign of severe pneumonia, this represented a small but important group to capture in the surveillance system. Children with oxygen saturation less than 90%, irrespective of respiratory rate, were thus also considered eligible for enrolment into the surveillance.

The original and the modified surveillance case definitions are outlined in Table 2.

**Table 2:** Pneumonia surveillance case definitions

Surveillance case definition in use in the WHO-supported system	Revised case definition introduced at commencement of enhanced system
Any child aged 2–59 months admitted to a hospital conducting surveillance, demonstrating:	A child (aged 2–59 months) is eligible for surveillance if they meet any one of the following:
cough OR difficulty breathing AND EITHER displaying fast breathing when calm (as defined by age): • > = 50 breaths per minute in infants aged 2–12 months • > = 40 breaths per minute in children aged 1–4 years	cough OR difficulty breathing, AND respiratory rate > =50 bpm (all ages), OR oxygen saturation < 90%
OR with a clinical diagnosis of severe pneumonia (based on the presence of chest indrawing, stridor, or a danger sign [inability to feed, vomiting everything, convulsions, prostration/lethargy]).	OR with a clinical diagnosis of severe pneumonia

The revision to the surveillance case definition did not alter the clinical management of patients.

#### Standardised recording of clinical signs

Clinicians from all hospitals participated in multiple refresher training sessions on the standardised recording and collection of clinical signs (for example, counting respiratory rate for a full minute), to reduce inter-observer and inter-hospital differences in approach. Standardised pulse oximeters with paediatric sensors were provided to all participating hospitals to enable accurate measurement and recording of oxygen saturation.

#### Collection of risk factor information

An additional questionnaire collecting information on risk factors for carriage and disease, including household crowding, household smoke, number and PCV13 status of other young children in the house, and breastfeeding, was introduced.

#### Strengthening engagement with hospital surveillance teams

A dedicated surveillance team was established in each hospital, including key medical and nursing staff, representation from the hospital management, laboratory managers and hospital epidemiologists. This team oversees the coordination of surveillance in the hospital and is the liaison point for the PCV impact evaluation team. Hospital teams have regular (at least monthly) meetings with the PCV impact evaluation team. PCV impact evaluation team members also visit the sites weekly to monitor completeness of enrolments and completeness of data on CRFs. The PCV impact evaluation team includes a Principal Investigator, Epidemiologist, Project manager, Data manager and Laboratory manager.

With enhanced surveillance commencing in April 2015 and Phase 1 districts commencing vaccination in June 2016 we have been able to collect strong baseline data, with 14 months of enhanced surveillance pre-vaccine data prior to PCV13 introduction into these districts, and prospective collection of over 2 years of pre-vaccine enhanced surveillance data in the Phase 2 districts. Enhanced surveillance data will be collected for a minimum of 2 years from vaccine introduction into Phase 1 districts.

### Case identification and enrolment

Hospital doctors working in the admissions area of each hospital have been trained on the surveillance procedures, including application of the modified case definition and informed consent. Every child aged 2–59 months who is admitted with suspected pneumonia is assessed by a hospital doctor to determine whether they meet the surveillance case definition. Any child meeting the case definition is allocated a unique surveillance identification number and enrolled into the surveillance system. Children re-admitted within 14 days after a previous pneumonia admission and children residing outside the four evaluation districts are not eligible for enrolment into the enhanced surveillance.

The IB-VPD system is a public health surveillance system and as such does not require individual informed consent. As the additional items undertaken in the enhanced system are to serve a research objective, and include the collection of information and specimens that are not related to the clinical care of the patient, individual informed consent is required. The additional data and sample collection is only undertaken if the parent or guardian provides informed written consent.

Samples are collected as soon as possible after admission, ideally before treatment commences, and not after 72 h from time of admission. The date and time of admission and of collection of each specimen is recorded on the CRF. If hospital treatment (for example, a dose of antibiotics) commences prior to collection of samples, this is also recorded on the CRF. Any patients who are missed at time of admission are enrolled retrospectively into the surveillance and the CRF is completed. If the enrolment is within 72 h specimens are collected.

### Specimen handling, transportation and storage

Nasopharyngeal samples are collected and stored according to WHO guidelines [37]. Swabs are collected by nurses trained in this technique by the PCV impact evaluation team. After collection, nasopharyngeal swabs are stored in a refrigerator in the specimen collection area at each hospital. They are transported in a cool-box to the NCCD microbiology laboratory, where they are vortexed. Three aliquots are taken for each swab and frozen at ultra-low temperature (ULT, − 70 °C), along with the original sample tube, within 8 h of collection. Two aliquots of samples are then shipped in two separate shipments on dry ice to the MCRI laboratories in Melbourne where they are examined for pneumococcal content by qPCR and molecular serotyping conducted by microarray [38]. The RSV and influenza detection will be performed at NCCD laboratories using validated qPCR assays [32–35]. Testing for CRP is undertaken at the NCCD laboratories, and is measured quantitatively using an auto-analyser (Cobas Integra 400 plus, Roche, USA).

### Data entry and management

Completed CRF and consent forms are delivered to NCCD. All documents are checked and consent forms are securely archived in a separate area to the CRFs. There is direct follow up with hospitals by telephone in the case of missing, implausible or unclear values, and if required the CRF is returned to the hospital for correction by the relevant individual. A paper-based log of all queried and corrected values is maintained by the PCV impact evaluation team. An electronic register of PCV13 dose administration was developed for use in the immunisation clinics [39]. Information recorded on the CRF regarding the case’s PCV13 status (ideally documented by direct reference to the patient-held immunisation card, or if not available by parent report) is validated against the PCV13 immunisation database. Conflicting records are verified against the clinic’s hard copy immunisation record, and a log kept of any corrections made.

Data are double entered into a Microsoft Access database by PCV impact evaluation team personnel at NCCD. Data checking and cleaning is undertaken jointly between the NCCD and MCRI-based teams. Consistency checks between the double-entered databases are performed in Stata (College Station, TX, StataCorp LP) on a monthly basis. The team investigates inconsistencies between the databases by reviewing the original source CRF. An electronic log of all inconsistencies and corrections is maintained.

Chest radiographs are digitised and uploaded to an online REDCap database [40] hosted at MCRI, which is then remotely accessible by the project radiology experts, who review according to WHO criteria and enter the result directly into the database. Radiographs are re-read by two radiology experts. Inter-rater reliability will be assessed at various times during the project, with both radiologists reviewing a random sample of images and the results compared.

### Monitoring

A number of systems have been implemented to monitor completeness of enrolment and data quality.

The hospital epidemiologist reviews the paediatric admissions book on a daily basis to verify how many of the age-eligible suspected pneumonia admissions have been enrolled into the surveillance. The medical record of those that have not been enrolled is reviewed to verify that the patient does not meet criteria for enrolment. A log is kept to keep track of patients who decline consent to participate in the enhanced surveillance.

Each hospital submits a weekly report detailing the total number of pneumonia admissions and the proportion that were enrolled in surveillance. While recognising that not all pneumonia admissions will meet the criteria for surveillance, this statistic is an indicator that hospitals are able to rapidly and regularly report, enabling trends in enrolment to be monitored. Information is also reported on the number of patients meeting criteria that are not enrolled and reasons for non-enrolment.

Periodic monitoring of data on key clinical indicators such as respiratory rate and oxygen saturation is undertaken. Monitoring of these indicators provides insight into whether the measurement and recording is being done correctly. For example, clustering of recordings around certain values, such as respiratory rates clustered around 50 breaths per minute or oxygen saturation around 90%, suggests that refresher training on the measurement of clinical signs may be required. Delays in data entry experienced during the first year of the project limited this component of routine monitoring to a quarterly exercise, but thereafter it has been our aim to produce and review these summary statistics on a monthly basis.

To continuously monitor and enable feedback on radiology quality, each week hospital radiologists provide one or two of the films they have taken during that week that they would consider to be of high quality. The expert radiologist reviews and provides feedback on these films to the radiologists. The quality of the images is also continuously assessed as part of the standardised reading of the x-rays.

### Sample size

There are expected to be about 4500 pneumonia admissions among 2–59 month old children meeting case definition for surveillance annually across the four evaluation districts in the absence of PCV13, of which we estimated 18% would be radiologically confirmed. This equates to approximately 800 radiologically confirmed cases per year in the four evaluation districts, an incidence of 6 per 1000 population (based on 2015 population estimates of 124,168). A 25% reduction in radiological pneumonia is anticipated with the introduction of PCV13. Based on the anticipated number of radiologically confirmed admissions we will have at least 90% power to detect a significant difference in the radiological pneumonia incidence rate at the 0.05 level.

### Statistical analysis

The primary analysis statistic will be the radiological pneumonia incidence rate ratio (IRR) among 2–59 month olds comparing the pre- and post-vaccine periods, using data from both the Phase 1 and Phase 2 districts. The IRR will be estimated using a generalised linear mixed model, with fixed effects for PCV13 introduction and time period and a random effect for cluster (district). A Poisson regression model will be used (or a negative binomial model if there is over-dispersion), and the logarithm of the population denominators will be included as an offset variable. The coefficients of the model (i.e. fixed effect for PCV13) will be exponentiated to obtain IRRs with 95% confidence intervals. Population denominators will be obtained from the Mongolian Ministry of Health.

### Carriage surveys among healthy children

Cross-sectional carriage surveys in the two Phase 1 districts are undertaken in 1000 healthy children, with a representative sample of 500 children aged 12–23 months and 500 young infants aged 5–8 weeks old. The baseline survey was done prior to PCV13 introduction in 2015, and a further two surveys will take place following PCV13 introduction. Following informed consent, risk factors for carriage are recorded, as follows: PCV13 status, breastfeeding status, type of delivery (caesarean or vaginal), number of children aged under 5 years living in the household, and exposure to indoor smoke from cigarettes and stoves. A nasopharyngeal swab is taken, transported, stored and analysed according to the same methods used for the swabs collected from hospitalised pneumonia patients. All surveys are conducted at the same time and location each year, to account for any seasonal and geographical variations.

#### Participant identification and enrolment

Participants are recruited from a sample of immunisation posts and health centres in the two districts that were first to introduce PCV13. Sites were randomly chosen to be representative of the socio-economic spread across the different sub-districts in each of the Phase 1 districts. Health centre staff assist with identifying children who may meet eligibility criteria. Healthy children presenting for routine check-up aged 5–8 weeks and 12–23 months whose families have lived in the area for at least three consecutive months are eligible. Any child who has a temperature of > 37 °C per axilla is excluded. Study nurses explain the study purpose to the parent or guardian, verify eligibility, obtain informed consent and collect the information and swab from children for whom consent is given.

#### Sample size

A sample size of 281 in each survey has 90% power at a 5% significance level to show ~ 50% reduction in VT carriage prevalence (16 to 8%). As the true prevalence of VT carriage in this population is unknown, the sample size for each survey has been increased to 500 in the 5–8 weeks old infants and 500 for the 12–23 month group.

#### Statistical analysis

The prevalence of VT, NVT and all pneumococcal carriage will be documented for both age cohorts, broken down by year and, where possible, PCV13 vaccination status. Comparisons of median ages and other quantitative measures of participant characteristics from year to year, and by PCV13 vaccination status will be made using Wilcoxon rank-sum tests. Comparisons of sex, ethnic group distribution, and other categorical variables will be made using a Chi-square test. To examine the impact of PCV13 on carriage, we will calculate prevalence ratios that compare carriage in the pre-vaccine period with carriage in the first and final post-vaccine survey. To account for potential confounders, adjusted prevalence ratios will be calculated using log-binomial regression.

### Current status

The study is ongoing and expected to continue to 2020. Data analysis with publication of results is anticipated from 2019. As of 1st May 2018, we have enrolled 11,010 eligible cases into the study since start-up. Table 3 shows the baseline characteristics of children recruited to May 2018.

**Table 3:** Baseline characteristics of cases enrolled into pneumonia surveillance programme, Mongolia, 2015–2018 (*N* = 11,010)

		n (%)
Year of recruitment	2015^a^	1901 (17)
	2016	4025 (37)
	2017	3533 (32)
	2018^b^	1551 (14)
Age group	< 12 months	4107 (37)
	12–23 months	3376 (31)
	24–59 months	3527 (32)
Gender	Male	5914 (54)
District	Songinokhairkhan	2975 (27)
	Sukhbaatar	2350 (21)
	Bayanzurkh	3413 (31)
	Chingeltei	2272 (21)

^a^From 1st April 2015; ^b^To 1st May 2018

## Discussion

Establishing a robust surveillance system is an important component of monitoring the impact of PCV within a country. The enhanced surveillance system in Mongolia will facilitate the assessment of PCV13 impact on pneumonia, with radiological confirmed disease as the primary outcome. Establishing the surveillance system was not without its challenges, but the lessons learned in addressing these challenges may be of relevance to other countries seeking to establish surveillance for pneumonia.

There have been four key lessons learned thus far. The first lesson has been the importance of establishing a core group of hospital staff at each site to be responsible for the surveillance activities, and for the PCV impact evaluation team to work closely and collaboratively with each hospital surveillance team. The hospital teams should be active partners in the evaluation activity. Such partnership supports the collection of high quality data and ensures that any problems can be identified and addressed rapidly. The winter of 2015/16 in Mongolia was very severe, and hospitals were finding it difficult to manage surveillance enrolment with such a high patient load. In some hospitals, staff from other wards were brought in to relieve the burden in the admissions and paediatric wards, but several of these staff had not been trained on how to enrol patients into the surveillance system. It was not until later, when we reviewed the declining enrolment rate, that these issues were identified. We have since increased our focus on having more regular engagement with hospital teams and actively encourage teams to share with us their experiences in implementing the surveillance.

The second lesson is that a number of external factors can potentially influence disease burden estimates, by influencing the volume of patients presenting to hospital and their severity of disease. It is necessary to maintain awareness of such factors, and to implement strategies to be able to quantify any impact on surveillance-related outcomes. A significant external factor we have identified in this study is a change in referral practice. When our enhanced surveillance commenced, antibiotics included in the IMCI algorithms were available free-of-charge for inpatients only. In 2016, these antibiotics became available at no cost for outpatients presenting to Family Health Centres, which almost certainly would have resulted in a larger proportion of patients being treated as outpatients rather than being referred to hospital. Similarly, the 2013 revision of the WHO IMCI guidelines for the management of acute respiratory infection (removing chest indrawing as a sign of severe pneumonia requiring hospitalisation) was formally adopted in Mongolia in 2015. The adoption of these revised guidelines during the evaluation period would likely result in a reduction in the number of patients being referred for hospital care. The effect of these changes will be reviewed. Having mechanisms that enable regular communication and cooperation with key agencies, both within and outside of health, can help to ensure that potential external factors are identified.

The third lesson is that flexibility in data collection and processes is also necessary, in order to respond to changing circumstances. During 2015–2016 there was a large outbreak of measles in Mongolia. Cases emerged from March 2015, and by the end of that year it appeared that the epidemic was ending; however, in early 2016 there was a major resurgence that continued until July. As measles substantially increases the risk of pneumonia [41], there is potential for this outbreak to result in an increase in pneumonia cases, thus complicating our measurement of PCV13 impact. To be able to quantify the number of potentially measles-associated pneumonia cases being captured in the surveillance system, it was necessary to modify the CRFs and conduct a training update with hospital staff in order to collect data on the measles history of each surveillance case.

The fourth lesson is to ensure a consistent application of the pneumonia surveillance case definition. It is challenging to develop and apply a clear case definition that achieves a balance between capturing all cases that are likely true pneumonia, without overloading doctors with enrolment of all patients with acute respiratory infection, the most common cause of hospital admission for children in Mongolia and much of the world. A review of the existing IB-VPD surveillance system in early 2015, just prior to the commencement of enhanced surveillance, identified the problem of under-enrolment. Consultation with hospital staff identified a lack of clarity in the surveillance case definitions. In some hospitals, suspected pneumonia cases reporting prior antibiotic use (e.g. at home) prior to admission were also being excluded unnecessarily from the surveillance system (prior antibiotic use is recorded on the CRF, but not an exclusion criterion). In liaison with the Ministry of Health and WHO, the surveillance case definition was updated to be more specific (thus reducing the number of eligible cases and consequently the case load for admitting doctors). While this was an important step to ensure all cases could be captured, a shortcoming of this change to the definition is that it will be more difficult to compare results of impact on clinical pneumonia with those from the earlier clinical trials. To ensure that all hospital staff were using the correct case definition and maintain consistency in enrolment from the start of the enhanced surveillance, refresher training in the case definition was conducted in all hospitals. A number of printed resources were produced to assist hospital staff in correct application of the simplified surveillance case definition and to ensure that all necessary surveillance data are collected. This included a wall poster to be placed in the relevant areas in the hospitals, and easy-reference pocket guides to be carried by the doctors. The weekly enrolment rate monitoring system described above was also established. Frequent staff turnover in hospitals presents the need for frequent re-training, both in the case definition and in surveillance procedures. Strategies that we have implemented to meet this need for ongoing training include the train-the-trainer approach and production of short instructional videos.

Given the concern about correct application of the case definition and cases being missed, to ensure complete ascertainment of eligible cases for the first year of enhanced surveillance, a medical record review of all age-eligible pneumonia admissions to the 5 participating hospitals has been undertaken for the first year of enhanced surveillance. The culture of detailed medical record keeping in Mongolia has allowed us to retrospectively capture the majority of data from missed cases through review of their patient file, though unfortunately with the notable absence of radiology data. In addition a retrospective review will also be done for all pneumonia cases in children < 5 years from April 2013 to March 2015, and all empyema cases in children < 5 years from April 2010 to March 2015.

Radiological pneumonia has been shown to be the best pneumonia endpoint for pneumococcal disease [15–18]. Radiology is therefore central to this evaluation, but it is an area in which many resource-constrained settings have limited scope to produce reliable, high quality data. In establishing the data collection systems for this evaluation, the PCV impact evaluation team worked very closely with hospital radiology departments to strengthen and support their systems, equipment and capacity. A comprehensive audit of equipment and processes as well as training was done prior to start-up of enrolment. These actions and the ongoing weekly quality assurance checks, has resulted in the production of high quality radiologic films for the study. Fewer than 3% of the 3100 films that had been re-read by the end of 2016 were uninterpretable for endpoint consolidation. This is a lower proportion than that reported in the PERCH study (10%) [42]. Ensuring adequate resources are available for ongoing radiology equipment maintenance and repair should be considered in developing evaluation budgets.

Virology testing is a novel aspect of this evaluation project because the clinical evidence in the context of pre- and post-PCV introduction is still limited. The information provided from this study will be critical not only for Mongolia but for many other countries as RSV is a leading pathogen in paediatric LRTI worldwide. These data will also provide a valuable baseline of seasonal, epidemiological burden of RSV and influenza infections in children less than 2 years of age with severe pneumonia. This will complement data collected by the Ministry of Health and WHO RSV surveillance system, which began in May 2017 as part of the influenza-like illness programme and detects milder cases of RSV in all age groups. Moreover, laboratory capacity building on RSV and influenza testing derived from this project has provided the NCCD Virology Laboratory with additional RNA extraction and qPCR machines and a higher capacity to deal with the high volume of samples derived from the project, as well as routine testing, and from the RSV and influenza surveillance. Following each qPCR run, results are loaded into a cloud account that can be accessed by all collaborators. The surveillance may have missed some children with RSV as the enhanced surveillance system did not enrol children with bronchiolitis and the oxygen cut-off was lower than that used for RSV [29].

While pneumococcal nasopharyngeal carriage is a precursor to pneumococcal disease, the interplay between the presence of pneumococci in the nasopharynx and pneumonia is poorly understood. High pneumococcal density (8000 copies/ml) from nasopharyngeal swabs has been shown to have high diagnostic accuracy in diagnosing pneumococcal pneumonia in adults [43]. In children, pneumococcal density in the nasopharynx is higher in children with radiological pneumonia compared to healthy controls or children with milder respiratory tract infection [44]. Nasopharyngeal swab analysis of pneumonia cases provide important information on likely pneumonia aetiology as well as additional information on the circulating serotypes in a population, and can assist in monitoring the direct and indirect effects of PCV13 introduction. To assess pneumococcal carriage we have applied gold-standard microbiological approaches. However, this strategy was also selected as the laboratory flow is relatively ‘modular’ facilitating strengthening of key assays such as *lytA* qPCR to support on-going surveillance, using the same DNA extraction and real-time PCR platforms as the virology assays. A phased strategy for long-term technology transfer has been developed with the project partners, and will continue to evolve as the project progresses. To date, activities have included a joint visit from the MCRI and IB-VPD microbiologist, an MCRI only visit and a laboratory training visit at MCRI for two NCCD laboratory personnel.

Finally, it is important to put mechanisms in place to ensure that the expanded surveillance and key laboratory capacity provided through the funded project can be sustained. For example, in the case of radiology, equipment was provided to some sites to enable the digitisation of x-rays. This is essential for the evaluation, as it enables the electronic archiving and transfer of images for re-reading and for archiving project data. In sites where this equipment could not be supplied, the project meets the cost of the x-ray film and printing reagents and an x-ray scanner is used to produce an electronic copy. However, in most hospitals there is no internal network that allows doctors to view the image on a computer elsewhere in the hospital, so it is impractical to only have an electronic copy of the radiograph. Without this internal network capability, and with very limited funds for printing x-rays, there is a risk that hospitals will revert to the earlier practice of fluoroscopy, the equipment provided will fall into disuse and the capacity that has been built among the local radiology teams to use the equipment will be lost. However, with the strengthened capacity for clinical research and surveillance created in country, due to exposure and participation of hospital staff, as well as the buy-in of the Ministry of Health to continue with the surveillance following the donor funding period, there is commitment to ensure that the programme is maintained.

Our study has a number of strengths. Firstly the methods used are consistent with other international research activities studying nasopharyngeal carriage, including studies undertaken by our group in Lao PDR and Fiji [45–47]. Secondly we used sensitive gold-standard molecular methods to detect pneumococcus in the nasopharynx. Our method of microarray with a culture amplification step was the top-performing method in a large, international multi-centre study exploring the best pneumococcal serotyping methods for carriage studies [31], and will provide density data to the level of serotype. Thirdly, the identification of radiological pneumonia cases that have an elevated CRP [48], and have either heavy nasopharyngeal carriage, or carriage of pneumonia-associated serotypes, will identify a subgroup that is more likely to be pneumococcal in origin and may thus be expected to decline in incidence more sharply than the total radiological incidence with the introduction of an effective PCV. Fourthly, our community carriage surveys include children too young to be vaccinated (5–8 weeks of age). This allows us to document the herd immunity in very young infants who are at high risk of pneumococcal disease, especially meningitis.

## Conclusion

The PCV impact evaluation in Mongolia will provide data to help guide national vaccine policy, and contribute to the limited evidence base on the burden of pneumococcal pneumonia in LMICs, particularly in Asia. In addition, it provides one of the first experiences of implementing prospective, population-based pneumonia surveillance to evaluate the impact of PCV in a resource-limited setting. With increasing introduction of PCV in these settings, the methods, experiences and lessons learned from this activity may be used to guide the development of such systems in other areas.

## Abbreviations

CRF: Case report form
CRP: C-reactive protein
GoM: Government of Mongolia
IB-VPD: Invasive Bacterial Vaccine Preventable Disease
IMCI: Integrated Management of Childhood Illness
IPD: Invasive pneumococcal disease
IRR: incidence rate ratio
LMICs: low and middle-income countries
LRTI: lower respiratory tract infection
MCRI: Murdoch Children’s Research Institute
NCCD: National Center of Communicable Diseases
NVT: Non-vaccine type
PCR: Polymerase chain reaction
PCV: Pneumococcal conjugate vaccine
PCV13: 13-valent pneumococcal conjugate vaccine
qPCR: quantitative PCR
RSV: Respiratory syncytial virus
SaO_2_: Arterial oxygen saturation
ULT: Ultra-low temperature
VT: Vaccine type
WHO: World Health Organization
